# Kneading-Dough-Inspired Quickly Dispersing of Hydrophobic Particles into Aqueous Solutions for Designing Functional Hydrogels

**DOI:** 10.3390/gels9030242

**Published:** 2023-03-18

**Authors:** Jun Huang, Youqi Wang, Ping Liu, Jinzhi Li, Min Song, Jiuyu Cui, Luxing Wei, Yonggan Yan, Jing Liu

**Affiliations:** 1 State Key Laboratory of Shale Oil and Gas Enrichment Mechanisms and Effective Development, Beijing 102206, China; 2 Key Laboratory of High Efficiency and Clean Mechanical Manufacture of Ministry of Education, School of Mechanical Engineering, Shandong University, Jinan 250061, China; 3 Research Institute of Petroleum Exploration and Development, Sinopec, Beijing 102206, China; 4 Oil and Gas Development Management Center of Shengli Oilfield Company, Sinopec, Dongying 257000, China; 5 Xinxing Cathay International (Beijing) Institute of Materials Technology Co., Ltd., Beijing 100078, China

**Keywords:** dough making, hydrophobic particles, polyethyleneimine, hydrogel, enhanced-mechanical strength

## Abstract

Hydrogels containing hydrophobic materials have attracted great attention for their potential applications in drug delivery and biosensors. This work presents a kneading-dough-inspired method for dispersing hydrophobic particles (HPs) into water. The kneading process can quickly mix HPs with polyethyleneimine (PEI) polymer solution to form “dough”, which facilitates the formation of stable suspensions in aqueous solutions. Combining with photo or thermal curing processes, one type of HPs incorporated PEI-polyacrylamide (PEI/PAM) composite hydrogel exhibiting good self-healing ability, tunable mechanical property is synthesized. The incorporating of HPs into the gel network results in the decrease in the swelling ratio, as well as the enhancement of the compressive modulus by more than five times. Moreover, the stable mechanism of polyethyleneimine-modified particles has been investigated using surface force apparatus, where the pure repulsion during approaching contributes to the good stability of the suspension. The stabilization time of the suspension is dependent on the molecular weight of PEI: the higher the molecular weight is, the better the stability of the suspension will be. Overall, this work demonstrates a useful strategy to introduce HPs into functional hydrogel networks. Future research can be focused on understanding the strengthening mechanism of HPs in the gel networks.

## 1. Introduction

Hydrogels are three-dimensional polymer networks that have received great attention because of their high-water contents and good flexibility [1]. Composite hydrogels with tunable chemical and physical properties are excellent candidates for bio-medical research [2,3], oil production [4], adsorbents for removing hazardous pollutants [5,6] underwater adhesive [7], and other engineering applications [8,9]. Polyethyleneimine (PEI) is a positively charged polymer with a repetition of the ethyleneimine motif in the molecule structure [10]. The chemical structure (linear or branch) of PEI has a strong influence on its property. Branched PEI that contains primary, secondary, and tertiary amino groups is a suitable crosslinking agent that is commonly used for developing functional hydrogels [11], polymer coatings [12], DNA complexation and transfection in vitro and in vivo [13]. 

Previous studies demonstrated that by introducing PEI into the traditional polyacrylamide (PAM) network, ultra-stretchable hydrogels with an extension ratio of over 30,000% were achieved at room temperature. Moreover, the PEI-incorporated composite hydrogel can be used as anti-fog/frost coatings [14], flexible dual-responsive temperature and strain sensors [15], reversible adhesives [16,17], and adsorbents for removing heavy metal ions (e.g., chromium) from wastewater [18]. As far as we know, there is insufficient research on the application of PEI for surface hydrophobicity treatment, especially for hydrogel synthesis.

The introduction of hydrophobic moieties into hydrogel networks can bring intriguing structural, physical, or chemical properties for engineering applications (e.g., hydrophobic drug carriers and flooding agents in oil production). Most previous studies rely on the utilization of surfactants when adding hydrophobic material into aqueous solutions. For example, Can et al. developed a sodium dodecyl sulfate (SDS) micelle-crosslinked hydrogels by copolymerization of hydrophobic monomer with hydrophilic monomer in an aqueous solution. The hydrophobic monomer was dissolved in the SDS micelle solution. The obtained hydrogel was self-healable and showed good mechanical properties [19]. Zhu et al. developed a strategy of adding hydrophobic polymer coatings with a hydrophobic oil layer onto the hydrogel surface, which greatly enhanced the stability and water retention capacity of the obtained hydrogel [20]. Hydrophobic latex particles prepared via emulsion polymerization have been added into hydrogel networks as crosslinking points. Hydrophobic interactions between those particles could effectively dissipate deformation energy and endow the hydrogels with enhanced mechanical strength [21]. Alternatively, using SDS and gum arabic as the combined surfactants, Gao et al. developed an approach of forming stable hydrophobic association centers, which led to the increase in fracture stress and toughness of poly(butyl acrylate) latex particles incorporated hydrogels [22]. 

Because of its versatile functionality and tunable size, SiO_2_ particles are considered suitable materials for introducing crosslinking points during hydrogel synthesis. Yang et al. proposed dual-crosslinked hydrogels through the copolymerization of hydrophilic acrylic acid and hydrophobic octylphenol polyoxyethylene acrylate on the surface of SiO_2_ particles. Hydrophobic interaction as well as molecular entanglement could enrich the energy dispassion points during loading. Therefore, the mechanical properties can be tuned by adjusting the content of SiO_2_, the weight of the polymer, and surfactants [23]. Furthermore, the same research group developed an approach of adding core–shell inorganic–organic hybrid latex particles (SiO_2_-g-poly(butyl acrylate)) as the hydrophobic crosslinking centers for improving the mechanical and self-recoverable properties of hydrogels [24]. In spite of the achievements in developing hydrophobic moieties-incorporated hydrogels, it is still challenging to evenly and rapidly disperse hydrophobic materials into aqueous solutions when fabricating hydrogels with desired properties. 

In this study, hydrophobic particles (e.g., polytetrafluoroethylene and silane-treated SiO_2_) are successfully incorporated into PEI/PAM composite hydrogels through a “kneading-dough” process followed by UV/thermal curing. Surface force measurements were conducted to understand the stabilization mechanism of polyethyleneimine when making precursor suspensions. Compression and rheology tests were conducted to understand the mechanical properties and the capability of self-healing behavior of the obtained hydrogels. The effect of adding hydrophobic particles on the swelling kinetics of the composite hydrogels has also been studied. Overall, this work proposes a facile strategy that particularly holds the capability of controlling the number of HPs in hydrogels, leading to enhanced mechanical strength as well as prolonged service lifetime in engineering applications.

## 2. Results and Discussion

### 2.1. Dough-Making of PEI@ HPs

**Figure 1:**
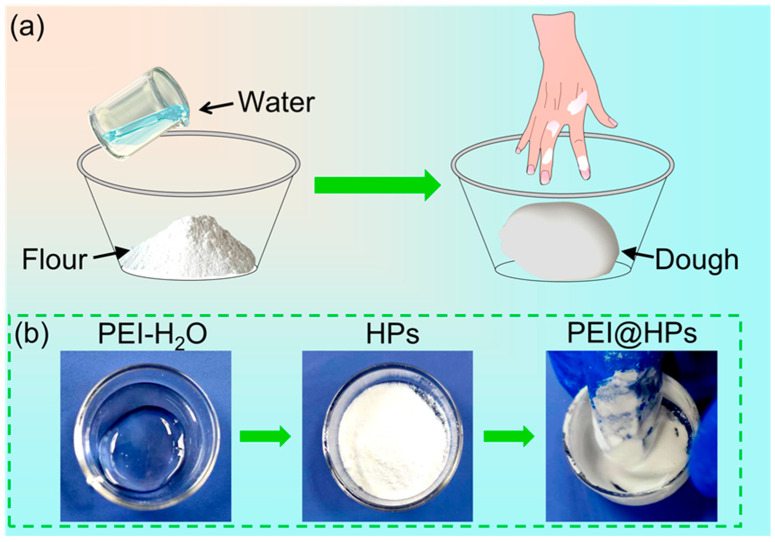
(**a**) Scheme of kneading dough with flour and water. (**b**) Photos of kneading HPs (e.g., KH570-modified SiO_2_) with PEI solution (Mw ~70,000, 50 wt%) until smooth and sticky dough-like gel was achieved.

The mixing step plays an important role in the dough-making process. As shown in Figure 1a, water is added to flour and then the mixture is stirred or kneaded to make viscoelastic dough. Through proper kneading for a certain period of time, the flour gradually absorbs water and the hydrated gluten proteins will form an extensible three-dimension network that is stabilized by disulfide bonds and non-covalent bonds [25,26]. As the dough is kneaded, mechanical energy is imparted to mix water with wheat flour, and the proteins line up to form giant chains of amino acids, creating a matrix within the dough itself [27]. The adsorption amount of water depends on the hydrophobicity of different ingredients such as protein (gliadin and glutenin), starch, and wheat polysaccharides (pentosans and β-glucans) [26]. 

Generally, it is difficult to directly mix water with hydrophobic powders. Inspired by the dough-kneading process, the method of adding polymers such as PEI to particles for improving the dispersion performance of hydrophobic particles in aqueous solutions is presented. As shown in Figure 1b, after continued pressing and kneading, the mixture of PEI and SiO_2_ HPs becomes sticky and smooth, and a dough-like gel mixture is obtained. Branched PEI is serving as the crosslinking reagent, forming a polymer network with hydrophobic moieties and trapping large amounts of water. Notably, PEI aqueous solution (50 wt%) is used in this experiment for achieving a smooth dough mixture.

### 2.2. Fabrication of PEI@HPs-PAM Hydrogel

**Figure 2:**
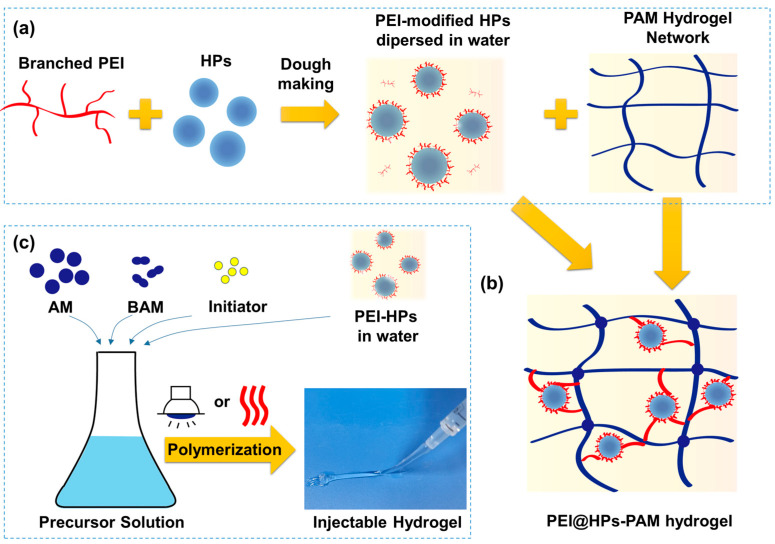
Schematic illustrations of (**a**) combining branched PEI, HPs, and PAM network and (**b**) injectable PEI@HPs-PAM hydrogel network. (**c**) Scheme of preparing PEI@HPs/PAM hydrogel via UV irradiation or heating.

Figure 2 shows the scheme of fabricating functional PEI@HPs-PAM composite hydrogel. Firstly, branched PEI dissolving in water (50 wt%) is mixed with HPs through the dough-making process (see Figure 1 and Figure 2a). Then the PEI-modified “dough” is dropped into the PAM-BAM-initiator precursor solution under continuous stirring. When the suspension is ready, the mixture is transferred into a cylindrical syringe followed by UV or heat curing. Then, the injectable hydrogel doping with hydrophobic particles is ready for further characterization (Figure 2b,c). 

**Figure 3:**
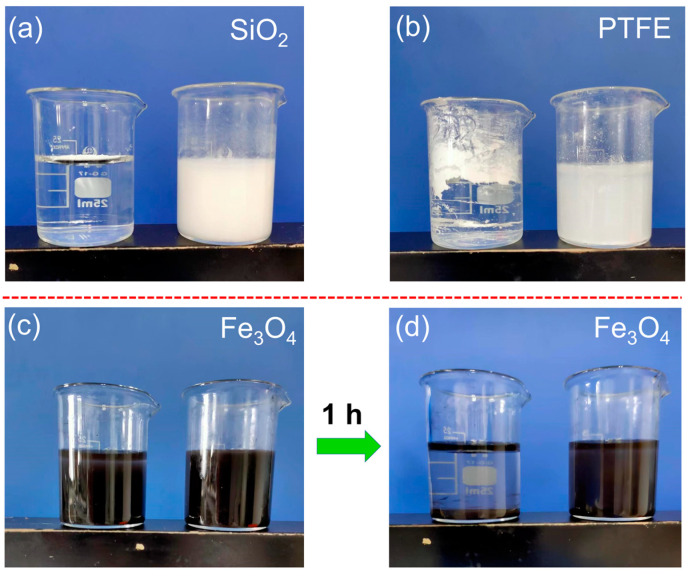
Comparison of the suspension effect for HPs and PEI-modified HPs. (**a**) Hydrophobic SiO_2_ particles (**left**) and PEI@SiO_2_ HPs (**right**) directly dispersed in water; (**b**) PTFE HPs (**left**) and PEI@PTFE HPs (**right**) dispersed in water. (**c**) Images of freshly prepared Fe_3_O_4_ particles (**left**) and PEI@Fe_3_O_4_ particles (**right**) dispersed in water. (**d**) Images of Fe_3_O_4_ particles (**left**) and PEI@Fe_3_O_4_ particles dispersed in water and settled for 1 h.

Dispersion tests of HPs and PEI-HPs-water dough in aqueous conditions were conducted to investigate whether PEI-modified particles could have better dispersion performance than the original ones. Two types of HPs including KH570-treated SiO_2_ and PTFE are used in this study. PEI-HPs dough is added to aqueous solutions under continuous stirring for 10 min. The left beakers in Figure 3a,b show that the particles without dough-making processing simply float on the water surface. However, PEI@SiO_2_ HPs are quickly dispersed into aqueous solutions in 2 min with the white milky suspension being generated. A similar phenomenon also occurs between PTFE HPs and PEI@PTFE HPs (Figure 3b). Importantly, the obtained suspension can keep stable for more than 24 h. To check the feasibility of this dispersion method on hydrophilic particles, hydrophilic Fe_3_O_4_ particles were also tested. Figure 3c,d show that the stirring-dispersed Fe_3_O_4_ particles are temporarily stable, most Fe_3_O_4_ nano-particles either gather on the water surface or cluster at the bottom of the beaker. By comparison, the gathering phenomenon of PEI@Fe_3_O_4_ NPs is not as obvious as that of using bare Fe_3_O_4_ nano-particles (see Figure 3d), demonstrating that the modification of PEI can prolong the stabilization time of nano-particles in water. Overall, no matter whether hydrophobic or hydrophilic, the modification of PEI polymer through the dough-kneading process could efficiently improve the dispersion performance of particles in water.

**Figure 4:**
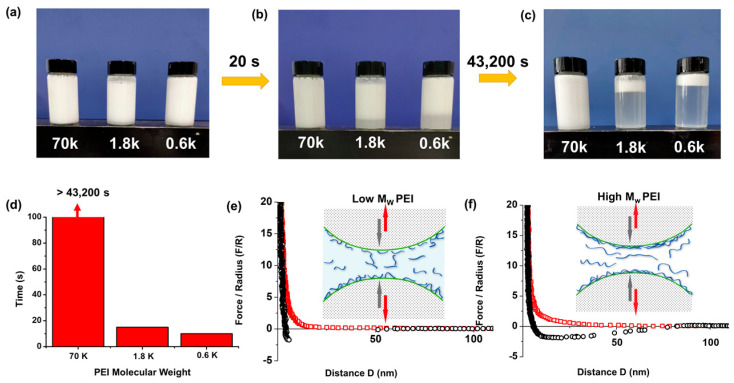
The effect of PEI molecular weight on the stability of HP suspensions. (**a**–**c**) Photos of SiO_2_ HPs modified with PEI polymer (molecule weight Mw = 600, 1800, and 70,000). (**d**) Stabilization time of SiO_2_@PEI suspensions prepared via PEI 600, 1800, and 70,000, respectively. (**e**,**f**) Force–distance profiles of mica–mica surfaces after injecting PEI at high (**e**) or low (**f**) molecular weights.

The molecular weight of polymer plays an important role in interfacial stability. In this work, PEI with different molecular weights (Mw = 600, 1800, and 70,000) were used during “dough” processing. As shown in Figure 4a, the HPs modified with PEI polymer can be dispersed into aqueous solutions forming milky suspensions. However, the suspensions show rather different stability. Figure 4b,c indicates that after keeping stationary for 20 s, the particle suspensions made from the dough mixing with PEI-1800 and PEI-600 start to rise and gather on top of solution surface. In addition, the suspension made with PEI-600 shows the highest rising rate. On the other hand, the HPs suspension made with high molecular weight PEI (Mw = 70,000) is stable for more than 43,200 s (Figure 4c,d). This test proves that the molecular weight of PEI plays a dominant role in stabilizing the particle suspensions. 

In order to further understand the stabilization mechanism of PEI-modified particles in water, surface force apparatus (SFA) was used to directly characterize the interaction forces. It is known that SFA can quantify the surface interactions between two surfaces across different aqueous conditions [28]. Figure 4e,f show the force–distance curves of two atomic smooth mica surfaces after injecting PEI solutions and stabilizing for 30 min. The testing results indicate that a thin layer of PEI film (<5 nm) was confined between mica surfaces, resulting in strong repulsion when approaching two mica surfaces. It is well known that amine-rich PEI has an isoelectric point of 10.8–11.0 [29], and those PEI chains are positively charged at pH ~7. Both mica and silica are negatively charged around neutral pH [29,30]; therefore, PEI will adsorb onto mineral surfaces and form a thin adsorbed layer due to the electrostatic interaction. This is also in consist with previously reported results that PEI could quickly adsorb onto silica surfaces when the pH is above 3, and when the concentration of PEI is high enough, it will cause charge neutralization and even charge reversal at high polymer concentrations [31].

As shown in Figure 4e,f, pure repulsion is measured when bringing the two smooth mica surfaces into contact after injecting the PEI solution. Additionally, the range of repulsion increases from ~20 nm to ~55 nm when the molecular weight of PEI increases by two orders of magnitude, which is related to the change in adsorption amount and the configuration of PEI chains. Herein, interactions such as electrostatic force and steric force play dominant roles when bringing the two surfaces into close contact. Meanwhile, when the two mica surfaces were separated from the contact state, small adhesion (~2.5 mN/m) was measured. According to previously reported results, the measured adhesive force is caused by the polymer bridging via hydrogen bond and possible charge attraction between silicate ions with the adsorbed PEI layers of the interacting particles [32,33]. Additionally, the adhesion measured for high molecular weight PEI shows obvious stretching, which is also a sign of bridging effects during separation. Generally, the interaction forces of two particles in aqueous solutions are highly dependent on the state of surface charge and the configuration of adsorbed PEI polymer chains on particle surfaces. In this situation, a higher molecular weight of PEI means a longer chain of PEI as well as a higher number of positive charges, demonstrating a longer range of repulsion together with a more complicated entangling status of PEI@HPs in solution. Therefore, HPs suspension made with higher molecular weight PEI can keep a long time of even particle distribution.

**Figure 5:**
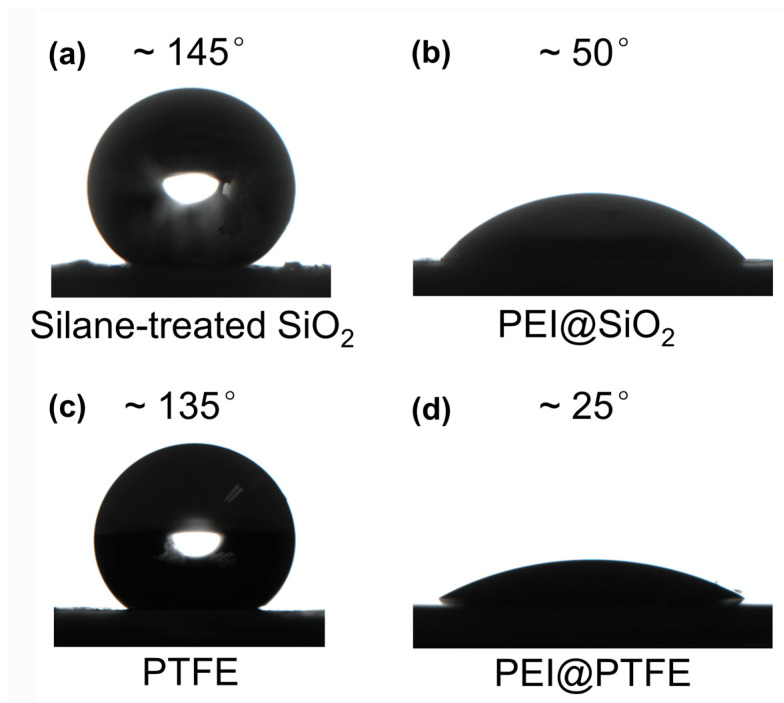
The water contact angles of (**a**) silane-treated SiO_2_ HPs, (**b**) PEI@SiO_2_ HPs, (**c**) PTFE HPs, and (**d**) PEI@PTFE HPs.

Water contact angle measurements were carried out to investigate the effect of PEI modification on the hydrophilicity of particles. As shown in Figure 5a, the water contact angle on silane-treated SiO_2_ HPs is 145°. In this case, PEI helps to diminish the surface energy mismatch between SiO_2_ HPs and the hydrogel precursor solution, which also facilitates the uniform distribution of hydrophobic SiO_2_ HPs. After modifying hydrophobic SiO_2_ HPs with PEI through the dough-making process, the water contact angle of PEI@SiO_2_ HPs decreases significantly to 50° (Figure 5b). Figure 5c shows that the water contact angle of PTFE HPs is 135°, also showing high hydrophobicity. However, after PEI modification, the water contact angle on obtained PEI@PTFE HPs dramatically decreases to 25°. Obviously, the water contact angle investigation of HPs and PEI-modified HPs stupendously proves that the adsorption of PEI on HPs surely changes the hydrophobicity of HPs, which is consistent with the above studies. The conversion of hydrophilicity of both SiO_2_ and PTFE HPs demonstrates the great potential of using PEI for surface functionalization. The “kneading-dough” method could provide a facile approach to fabricating functional hydrogels incorporating different amounts of HP.

**Figure 6:**
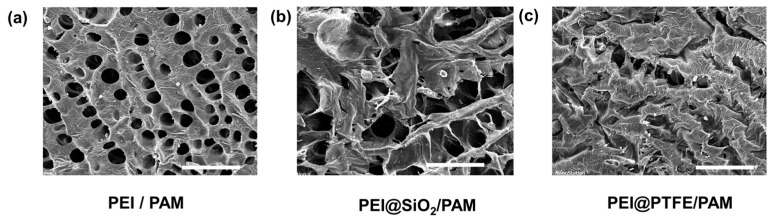
SEM images of freeze-dried (**a**) PEI/PAM (**b**) PEI@SiO_2_/PAM (**c**) PEI@PTFE/PAM hydrogels obtained through the dough-making process combined with UV curing, the scale bar is 100 μm.

The morphology of PAM hydrogel doping with different components (PEI, PTFE, SiO_2_) was characterized by SEM. Figure 6 indicates that all the obtained hydrogels present a three-dimensional porous network because of water loss during freeze-drying. The pore diameter is in the range of 20–30 μm for PEI/PAM hydrogel with a wall thickness of 40–50 µm. The introduction of SiO_2_ or PTFE HPs into the polymer network makes it difficult to observe the holes in the polymer network, which is likely caused by the easier collapsing of the dehydrated composites during freeze-drying. It is clear that HPs could be dispersed in the PEI/PAM hydrogel matrix and they forms a uniform network structure that can provide the hydrogels with excellent mechanical properties. 

### 2.3. Rheology and Self-Healing Properties of the PEI@SiO_2_/PAM Hydrogel

**Figure 7:**
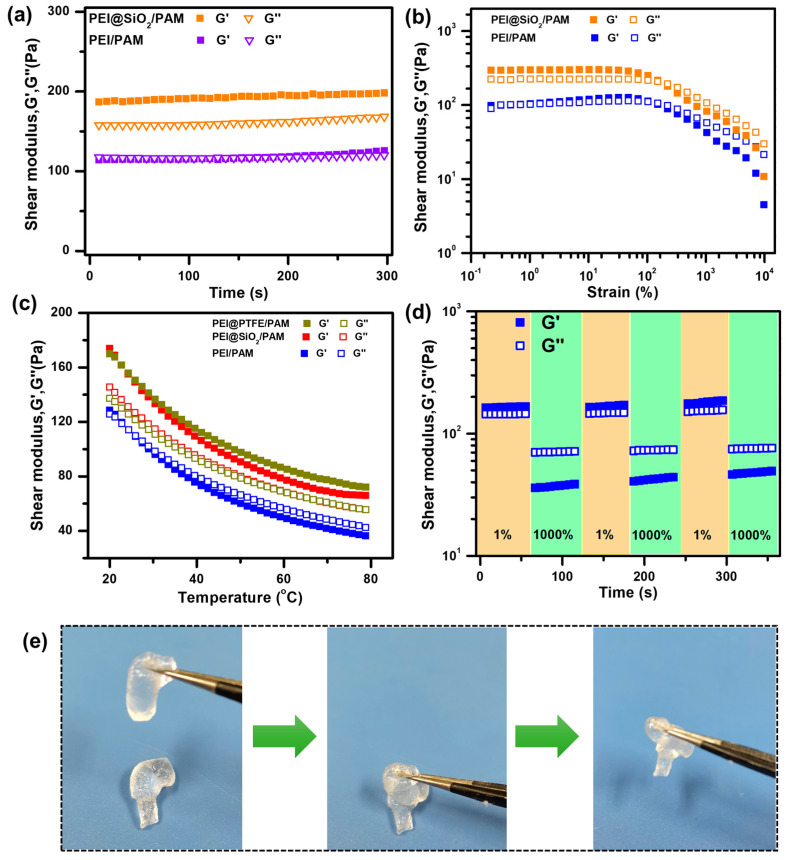
Rheology analyses of the PEI@HPs/PAM hydrogels. Storage modulus (G’) and loss modulus (G”) of (**a**) the hydrogel as a function of time at a fixed strain (*γ* = 1%) and a fixed frequency (f = 1 Hz). (**b**) Strain amplitude sweep (*γ* = 0.1~10,000%) at fixed frequency (*f* = 1 Hz). (**c**) G’ and G” versus temperature (*T* = 20~80 °C) at a fixed strain (*γ* = 1%) and a fixed frequency (f = 1 Hz). (**d**) G’ and G” versus time under alternated strain change cycles (1%→1000%→1%). (**e**) Photos demonstrate.

Rheology tests were conducted to measure the mechanical property of hydrogels. As shown in Figure 7a, the storage modulus (G’) curve of PEI@SiO_2_/PAM is above the loss modulus (G”) curve over the entire measurement range. For the case of PEI/PAM, the G’ value is much lower than that of PEI@SiO_2_/PAM. The difference in G’ suggests a higher degree of crosslinking of the polymer network after introducing SiO_2_ HPs. Moreover, upon 1% strain, the values of G′ and G” maintained steady with time, suggesting the great stability of PEI@SiO_2_/PAM hydrogel. Figure 7b suggests that G’ and G” remain unchanged when the applied shear strain is less than 200% at 25 °C, while both of them decreased rapidly with the continuous increase in shear strain from 200∼10,000%, demonstrating a shear-thinning property of PEI@SiO_2_/PAM hydrogel. The dramatic decrease in both G’ and G” with the continuous increase in shear strain from 200∼10,000% demonstrates that the mechanical property of PEI@SiO_2_/PAM hydrogel will deteriorate if the hydrogel experiences great pressure or shear force. In other words, the prepared PEI@SiO_2_/PAM hydrogel in this study possesses a good shear-thinning property.

Figure 7c shows the rheology testing results of G’ and G” as a function of temperature. It is noted that the shear modulus (both G’ and G”) decreases as temperature increases from 20 °C to 80 °C. In addition, the modulus of PTFE-doped hydrogel is the highest among the testing samples. It is worth mentioning that G’ is above G” throughout the entire temperature range for HP-doped hydrogels. However, for PEI/PAM hydrogel, G” becomes larger than G’ when the temperature is greater than 26 °C, indicating the hydrogel is acting like a liquid. Additionally, the value of G’ keeps larger than that of G” at the same temperature, which also proves that the structure of PEI@SiO_2_/PAM hydrogel is rather stable at relatively high temperatures, which ensures the wide applications of PEI@SiO_2_/PAM hydrogel under hot environment. 

The rheology results prove that the introduction of HPs into the hydrogel network results in a significant increase in mechanical strength. In addition, the fabricated hydrogel also shows good self-healing properties. Figure 7d shows the rheology results of the self-healing property of PEI@SiO_2_/PAM hydrogel. When the oscillatory shear strain takes steps from 1% to 1000%, both the values of G’ and G” of PEI@SiO_2_/PAM hydrogel suddenly decrease, which is the result of shear-thinning property of PEI@SiO_2_/PAM hydrogel. When the shear strain changes from 1000% to 1%, G’ and G” completely recover in a few seconds, demonstrating the quick recovery of the inner network. It is manifest that this recovery can be repeated, which suggests that the self-healing property of PEI@SiO_2_/PAM hydrogel is stable. Bulk self-healing tests of PEI@SiO_2_/PAM hydrogel were conducted to further investigate its self-healing property. As shown in Figure 7e, the bulk hydrogel is cut into two separate pieces, and then they are brought into close contact. After a short period of time, the two separate pieces could integrate into one single piece. The experiment indicates that separate PEI@SiO_2_/PAM hydrogel pieces can heal together by simply contacting, demonstrating the exceptional self-healing property of PEI@SiO_2_/PAM hydrogel.

**Figure 8:**
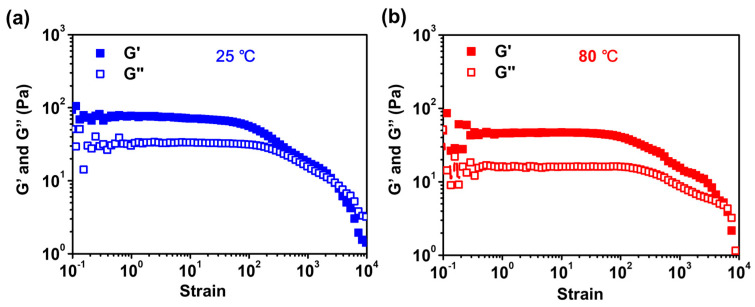
Comparison of the temperature stability of HPs doped hydrogel under varying shear strains. Storage modulus (G’) and loss modulus (G”) of the hydrogel on strain amplitude sweep (*γ* = 0.1~10,000%) at (**a**) room temperature (25 °C) and (**b**) high temperature (80 °C).

The hydrophobic particles can serve as crosslinking points and influence the thermal stability of hydrogel, and the change in strain under elevated temperatures is critical for PAM hydrogels used in oil production. Figure 8 shows the change in modulus for SiO_2_-doped hydrogels as a function of shear strain at 25 °C and 80 °C. It is noticed that the obtained hydrogel samples show good thermal stability at both temperatures. At 25 °C, G’ is larger than G” until the external strain reached 1000%, indicating the hydrogel network begins to break. Importantly, when the temperature reached 80 °C, the transition strain (G” > G’) increased to ~ 7000%, though the obtained G’ value decreases slightly as compared to that of 25 °C. The good thermal stability of the hydrogel can be potentially used for oil drilling processes, where high-temperature sustainability is required. 

### 2.4. Swelling of Hydrogels

**Figure 9:**
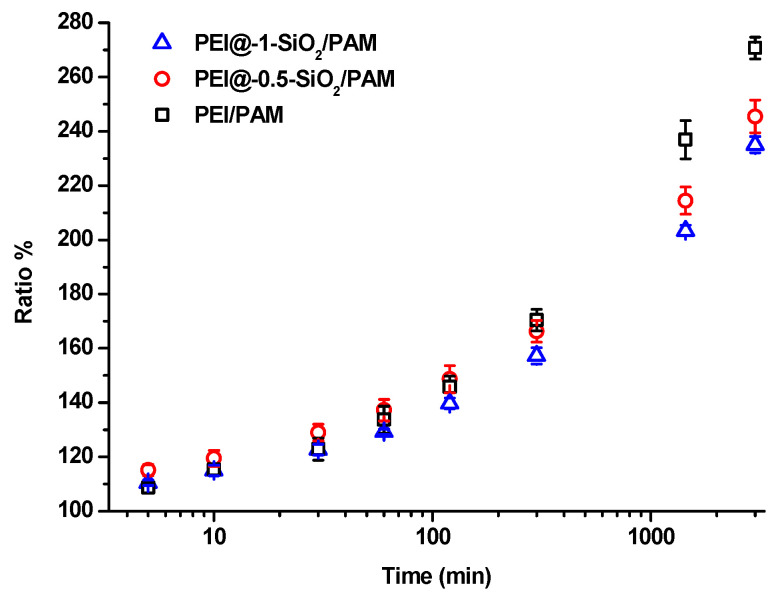
Swelling ratio of HPs-modified hydrogel samples (PEI/PAM, PEI@-0.5-SiO_2_/PAM, and PEI@-1-SiO_2_/PAM) as a function of immersing time, m_SiO2_:m_PEI_ = 0:1, 0.5:1 and 1:1, respectively. The swelling tests for each composition were repeated three times.

Previously reported results indicated that the hydrophobic SiO_2_ particles could serve as crosslinking points in hydrogel networks, which will influence the swelling kinetics of hydrogels [21,24]. Figure 9 shows the change in the swelling ratio of hydrogel samples at different time intervals. The mass of the hydrogels gradually increases because of the adsorption of water molecules. The swelling ratios of the samples are very close within the first 10 min, however, the swelling ratios of the PEI@SiO_2_/PAM hydrogel decreased obviously with increasing HPs content when the immersing time reached 3000 min (~30% difference between PEI@-1-SiO_2_/PAM and PEI/PAM). A possible reason is that the increase in crosslinking density of PEI@SiO_2_/PAM hydrogels resulted in a more compact network structure, which will slow down the adsorption and the diffusion of water molecules into hydrogels. 

### 2.5. Enhancing of Mechanical Properties

**Figure 10:**
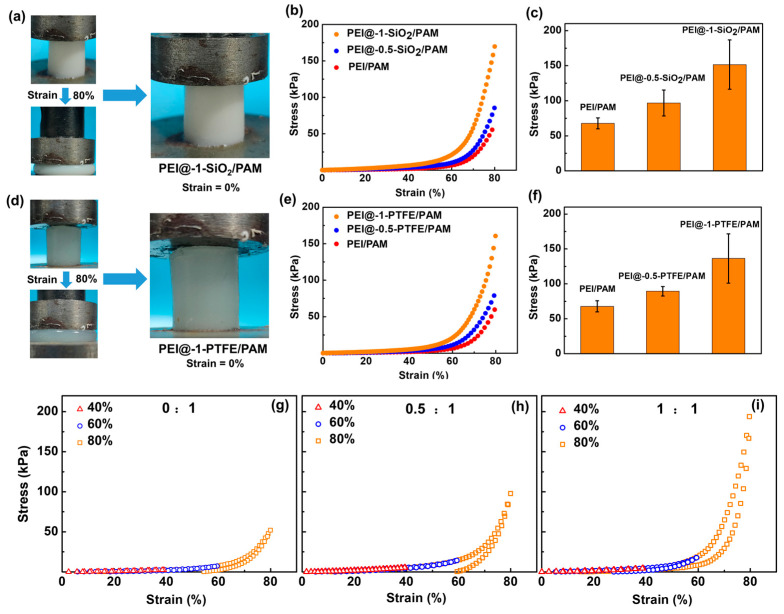
Compression test results of hydrogel samples with different components. (**a**) Photographs of PEI@-1-SiO_2_/PAM hydrogels under compression. (**b**,**c**) Compressive curves and compressive moduli of PEI/PAM, PEI@-0.5-SiO_2_/PAM, and PEI@-1-SiO_2_/PAM. (**d**) Photographs of PEI@-1-PTFE/PAM hydrogels under compression. (**e**,**f**) Compressive curves and compressive moduli of PEI/PAM, PEI@-0.5-PTFE/PAM, and PEI@-1-PTFE/PAM. (**g**–**i**) Loading–unloading curves of (**g**) PEI/PAM, (**h**) PEI@-0.5-SiO_2_/PAM, and (**i**) PEI@-1-SiO_2_/PAM. All compression tests were performed three times.

Inorganic materials are usually stiffer than hydrogels. It is anticipated that the introduction of HPs could alter the mechanical properties of hydrogels. Hence, the compressive stress as a function of strain for HP-incorporated hydrogels was measured. As shown in Figure 10a, the hydrophobic SiO_2_ particle-doped hydrogel shows good elasticity, which fully recovers after experiencing a compressive strain of 80%. When the compressive strain is lower than 40%, the slopes of the three samples are close (Figure 10b), indicating there is no significant difference in the compressive modulus at low compressive strains, which is similar to that reported by Hu et al. [34]. The compressive stress is greater than previously reported. However, the compressive strain increases quickly at large strains: the higher the amount of HPs is, the greater the slope will be. Statistic results of three individual testing samples prove that the maximum compressive stress increases monotonically with the increase in HP content (Figure 10c): the compressive modulus of PEI@SiO_2_/PAM hydrogel increased from 67.8 ± 7.9 kPa (m_SiO2_:m_PEI_ = 0:1) to 152 ± 35.7 kPa (m_SiO2_:m_PEI_ = 1:1). Similar findings are also observed for adding PTFE particles (Figure 10d–f), and the measured compressive stress at 80% strain also doubled as compared to that of PEI/PAM sample. Figure 10g–i show the loading–unloading curves of PEI/PAM, PEI@-0.5-SiO_2_/PAM, and PEI@-1-SiO_2_/PAM hydrogel samples under similar strain sweeps (0↔40%, 0↔60%, and 0↔80%). Mechanical hysteresis is observed for all three samples, suggesting possible energy dissipation of the polymer network during the loading–unloading processes. Because of the stiffing of the hydrogel network when increasing the amount of HP, the hysteresis (i.e., the area between the loading–unloading curve) clearly decreases. In addition, the subsequent loading curve almost overlaps with the ones at smaller strains, suggesting that the hydrogels could retain their outstanding performance of compression recovery after diverse strain sweeps. The mechanical testing results do prove that HP-doped PEI/PAM hydrogel samples exhibit tunable mechanical properties by changing the amount of HP, and the kneading-dough-inspired HPs processing is a universal approach for introducing HPs into hydrogel networks.

## 3. Conclusions

This work demonstrates a kneading-dough-inspired method of mixing hyper-branched PEI and HPs, which will facilitate the dispersion of HPs in hydrogel precursor solutions. Combined with photo or thermal polymerization, composite HPs-PEI/PAM hydrogels that are self-healable and mechanically tunable can be obtained. Surface force measurements indicate that the pure repulsion during approaching contributed to the good stability of the HPs in aqueous suspension. Additionally, the stabilization time is strongly dependent on the molecular weight of PEI. Rheology and compression tests prove that the dough-making strategy can achieve a controlled doping amount of HP in hydrogel networks, leading to the enhanced and tunable mechanical strength for engineering applications. It should be pointed out that due to the differences in hydrophobicity, the weight ratio of HPs to PEI solution cannot reach that of flour to water in a real dough-kneading process. In future studies, the strengthening mechanism of HPs incorporated into the hydrogel network will be further studied both from experimental and theoretical perspectives.

## 4. Materials and Methods

Acrylamide (AM) was purchased from Tokyo Chemical Industry (TCI, Shanghai, China). *N, N*-methylenebis(acrylamide) (BAM, purity 99%) and branched polyethylenimine (PEI aqueous solution, 50 *w*/*w*%, Mw~70,000), PEI (Mw 800, 1600, purity 99%) used during measurement were purchased from Aladdin Inc. (Shanghai, China) and Sigma-Aldrich (Shanghai, China). Hydrophobic silicon dioxide particles (SiO_2_ HPs, diameter ~20 nm, KH570 silane-treated) were purchased from Fuhong Materials Inc. (Shanxi, China). Muscovite mica sheets used for SFA measurement were obtained from S&J Trading (USA). Fe_3_O_4_ nano-particles (purity 99.5%) were purchased from Macklin Inc. (Shanghai, China). The conductivity of deionized water in this study is 0.05 μS/cm. All reagents in this work were used as received without further purification. For thermal initiation, ammonium persulfate (Macklin, Shanghai, China) was utilized to replace the photoinitiator, Irgacure 2959 (BASF, Shanghai, China). The weight composition of the materials is 8.5 g AM, 3.5 g PEI@SiO_2_, BAM 0.051 g, and 0.17 g ammonium persulfate and 55 g of water, and the weight ratio of HPs:PEI =0:1, 0.5:1 and 1:1, respectively.

### 4.1. Comparison of Dispersing SiO_2_ HPs and PEI@SiO_2_ into Deionized Water

Typically, PEI (50 *w*/*w*% aqueous solution) and HPs (m _SiO2_:m _PEI_ = 0.5:1, 1:1, m_PTFE_:m_PEI_ = 0.5:1, 1:1) were mixed to obtain PEI-modified SiO_2_ (PEI@SiO_2_) or PTFE HPs (PEI@PTFE). Then the modified particles were added to deionized water and stirred for 5–10 min, forming evenly distributed suspensions. To have a better comparison of the difference between SiO_2_ HPs and PEI@SiO_2_, SiO_2_ HPs were also added into equal amounts of deionized water and stirred for 10 min. Similarly, the dispersing effect of other particles (PTFE and Fe_3_O_4_) and PEI@particle was investigated as well.

### 4.2. Preparation of the PEI@SiO_2_/PAM, PEI@PTFE PAM Hydrogel

Polyacrylamide hydrogels (PAM) can absorb a large amount of water with insensitive response to pH or electrolytes, and their swelling capacity is not very sensitive to salt. Therefore, PAM is chosen as the model hydrogel for developing functional hydrogels. The PEI@SiO_2_/PAM hydrogel was prepared by using a UV or thermal curing method. PEI@SiO_2_ dough and suspension were fabricated according to the method mentioned above. Then, 8.5 g AM, 3.5 g PEI@SiO_2_, BAM (0.06 wt% of AM), and photoinitiator-2959 (2 wt% of AM, 0.732 mol% of AM) were dissolved in 55.5 g deionized water to obtain the hydrogel precursor solution. After degassing using the ultrasonication method, ultraviolet (UV) irradiation (365 nm, 10 W for 4 h) or thermal treatment (40 °C for 1 h) was conducted to cure the PEI@SiO_2_/PAM precursor solution. Then, the PEI/PAM hydrogels with other particles were fabricated as well. 

### 4.3. Scanning Electron Microscopy (SEM) Test

The structure of the hydrogel network was investigated by a field emission SEM (Keyence VK-X200, Itasca, IL, USA). The samples were prepared by freeze-drying at −56 °C for 48 h. After that, the samples were fractured and coated with a thin layer of gold film (<5 nm) for SEM characterization. 

### 4.4. Water Contact Angle Test

Hydrophobic SiO_2_ HPs and PEI@SiO_2_ were first dried in an oven to eliminate water in them. Then, the dried powders were pressed into flat thin sheets. Water droplets (~5 μL) were dropped onto the sheet surface. Additionally, water contact angles were measured using a contact angle meter (SDC-200S, Dongguan Shengding Precision Instrument Co., Ltd., Dongguan, China) at 25 °C. Similarly, the water contact angle tests of other particles were conducted as well.

### 4.5. Swelling Property Test

The weight variation rate of the PEI@SiO_2_/PAM hydrogel was used to investigate its swelling property. The hydrogel samples obtained after UV irradiation were weighed and quickly immersed in a large amount of water at room temperature. The weight of the sample was recorded by an electronic balance (Sartorius Scientific Instrument Co., Ltd., Beijing, China) at regular time intervals until it reached swelling equilibrium. The weight variation rate can be evaluated by Equation (1) as follows,

Swelling ratio (%) = *W*/*W*_0_ × 100% (1)

where *W*_0_ is the original weight of synthesized hydrogel and *W* is the measured weight of the swelling hydrogel at different time intervals.

### 4.6. Rheology Tests

Storage modulus (G’) and loss modulus (G”) of the PEI@SiO_2_/PAM hydrogel was investigated by a rheometer (MCR 302, Anton Paar, Austria) with a 25 mm cone plate. Strain amplitude sweep measurements (γ = 0.1~10,000%) were conducted at a fixed frequency of 1 Hz. Oscillatory frequency sweep measurements (f = 0.5~50 Hz) were performed at a fixed strain of 1%. G’ and G” versus time were tested at a fixed strain (γ = 1%) and frequency (f = 1 Hz). Modulus tests of the hydrogel at different temperatures were also conducted at a fixed strain (γ = 1%) and a fixed frequency (f = 1 Hz). The self-healing performance of the hydrogel was demonstrated by the continuous step strain at a frequency of 1 Hz, and the strains were switched from 1% to 1000% with a strain interval of 100 s.

### 4.7. Compression Tests

The compression tests of the hydrogel samples were measured by using a universal testing machine (ZLC-2D, Jinan XLC Testing Machine Co., Ltd., Jinan, China) at a loading rate of 10 mm/min with a 2000 N load cell. The testing materials were prepared by curing hydrogel precursor solution within a 10 mL syringe with a diameter of 15 mm and height of 12 mm.

### 4.8. SFA Tests

An SFA 2000 (SurForce LLC, Santa Barbara, CA, USA) system was used to measure the surface interactions of two solid surfaces after injecting PEI solutions. The detailed experimental procedure can be found elsewhere [9,35]. Briefly, two pieces of smooth mica surfaces were mounted onto the cylindrical disk in a cross-cylinder configuration, and then the PEI polymer solution (100 μL, 0.5 wt%) were injected in between the two surfaces. The system was equilibrated for 30 min before conducting force measurements. The SFA technique allows direct determination of interaction forces between two surfaces as a function of absolute separation distance with a high force sensitivity of 1 nN and the distance resolution down to 0.1 nm, and this technique has been extensively used to study the interactions in biological and un-biological systems [28].

## Data Availability

The data that support the findings of this study are available from the corresponding author, J.H., J.L., upon reasonable request.
